# Carbon Dioxide Insufflation in Routine Colonoscopy Is Safe and More Comfortable: Results of a Randomized Controlled Double-Blinded Trial

**DOI:** 10.1155/2011/378906

**Published:** 2011-06-15

**Authors:** M. Geyer, U. Guller, C. Beglinger

**Affiliations:** ^1^Gastroenterologie Wettingen, 5430 wettingen, Switzerland; ^2^Division of Visceral Surgery and Transplantation, Department of Surgery, University of Bern, 3010 Bern, Switzerland; ^3^Department of Gastroenterology and Hepatology, University Hospital Basel, 4031 Basel, Switzerland

## Abstract

Many patients experience pain and discomfort after colonoscopy. Carbon dioxide (CO_2_) can reduce periprocedural pain although air insufflation remained the standard procedure. The objective of this double-blinded, randomized controlled trial was to evaluate whether CO_2_ insufflation does decrease pain and bloating during and after colonoscopy compared to room air. *Methods*. 219 consecutive patients undergoing colonoscopy were randomized to either CO_2_ or air insufflation. Propofol was used in all patients for sedation. Transcutaneous CO_2_ was continuously measured with a capnograph as a safety parameter. Pain, bloating, and overall satisfaction were assessed at regular intervals before and after the procedure. *Results(data are mean *±*SD)*. 110 patients were randomized to CO_2_ and 109 to room air. The baseline characteristics were similar in both groups. The mean propofol dose was not different between the treatments, as were the time to reach the ileum and the withdrawal time. pCO_2_ at the end of the procedure was 35.2 ± 4.3 mmHg (CO_2_ group) versus 35.6 ± 6.0 mmHg in the room air group (*P* > .05). No relevant complication occurred in either group. There was significantly less bloating for the CO_2_ group during the postprocedural recovery period (*P* < .001) and over the 24-hour period (*P* < .001). Also, patients with CO_2_ insufflation experienced significantly less pain (*P* = .014). Finally, a higher overall satisfaction (*P* = .04 ) was found in the CO_2_ group. *Conclusions*. This trial provides compelling evidence that CO_2_ insufflation significantly reduces bloating and pain after routine colonoscopy in propofol-sedated patients. The procedure is safe with no significant differences in CO_2_ between the two groups.

## 1. Introduction

Many patients experience pain and discomfort after colonoscopy. An explanation for this observation is the retention of gas in the colon, as several liters of air are insufflated during colonoscopy. For decades, CO_2_ insufflation has been routinely used to create the pneumoperitoneum in lapraroscopic surgery. Conversely, room air insufflation has remained the standard of care in most endoscopy centres [1]. Preliminary studies indicate that insufflation of carbon dioxide (CO_2_) may reduce periprocedural pain. CO_2_ was first recommended 1953 to avoid gas combustion in the colon during electrocoagulation [2]. In 1986, the rapid absorption of CO_2_ in the colon and minimal interference with colonic circulation were described, therefore minimizing the risk of bowel ischemia [3]. CO_2_ is absorbed about 150 times faster compared to nitrogen and is rapidly eliminated through the lungs [4]. Interestingly, 30 minutes after insufflation with CO_2_, the gas has disappeared, whereas patients with standard room air insufflation still have a significant distension of both small bowel and colon [5]. Initial studies with a limited number of patients have suggested potential benefits for CO_2_ use: Sumanac et al. [6] examined 97 patients undergoing colonoscopy with either CO_2_ or room air insufflation and showed that 45% of the patients examined with room air had pain one hour after examination compared with only 9% in the CO_2_ insufflation group. Six hours after colonoscopy, the fraction of patients with pain was 31% in the room air group versus 7% in patients with CO_2_ insufflation. Conventional X-rays revealed colonic distension of more than 6 cm in diameter in 71% of patients assigned to room air compared with only 4% in the CO_2_ group [6]. Similarly, in the NORCCAP, a Norwegian colorectal prevention study [7], 267 patients underwent colonoscopy with insufflation of either room air or CO_2_, with the latter group experiencing less postprocedural pain. With recent new developments that facilitate the use of CO_2_, more data have become available supporting the observation that insufflation with CO_2_ causes less pain [8–10]. The same conclusion was drawn in the review of Dellon et al., which included 8 randomized controlled trials (RCTs) with two RCTs showing decreased flatus and 3 decreased bowel distension on abdominal radiography [11].

A large, population-based survey based on 7,370 colonoscopies performed in Norwegian endoscopy centers revealed that up to 24% of patients experience severe pain during colonoscopy [12]. According to one study, 20% of patients need more than two days before being able to return to their normal activities after screening colonoscopy [13]. These findings demonstrate that there exists a tremendous potential for improvement towards painless colonoscopy. 

In addition to better periprocedural pain control, another potential benefit of CO_2_ use is that no gas aspiration is necessary during withdrawal due to the fast gas absorption. Better colonic insufflation may be associated with a better diagnostic yield and especially a higher polyp detection rate. Comfort during and after colonoscopy represents a major issue for patient tolerance and acceptance. It is imperative that the nowadays recommended longer withdrawal times, and consecutively longer insufflations, do not compromise patient comfort. It would be wrong to shorten the withdrawal time during colonoscopy to make concessions with respect to patient comfort. 

The objective of the present randomized controlled double-blinded trial was to assess patient satisfaction, pain, and bloating between patients undergoing colonoscopy with CO_2_ insufflation versus standard room air.

## 2. Patients and Methods

In this randomized controlled trial, 219 consecutive patients undergoing colonoscopy in a private Swiss gastroenterology practice were enrolled between April 2008 to June 2008 (only one patient did not participate in this time period). Patients were randomly assigned to colonoscopy with either CO_2_ versus room air insufflation. This randomized trial was double blinded, as neither the patients nor the gastroenterologist were aware of the intervention. The randomisation was done with a dice for each patient. If the number was even, the patient underwent colonoscopy with CO_2_, if the number was uneven, colonoscopy with room air. The study nurse, without any involvement of the investigator, did the selection. The ethical committee approved the trial, and written patient consent was obtained for each patient for colonoscopy and for the study receiving patient study information including an informed consent section on a visit prior to the scheduled colonoscopy. 

All patients that seemed medically fit for an ambulatory colonoscopy were enrolled in this study. No particular exclusion criteria were used to maximize the generalizability (external validity) of this study. All colonoscopies were done by the first author and carried out with standard Pentax endoscopes (EC-3885K and EC-380FKp) with a standard processor EPK 1000. For CO_2_ insufflation, the medical licensed and approved CO_2_-Efficent Insufflator device (EZEM company Westbury NY 11590, US) was used. This insufflator is connected to a 10 L CO_2_ bottle. The CO_2_ is then supplied over a tubing set connected with a branch connection to the water bottle tube directly connected to the endoscope. The flow rate (basal flow rate 0.5 L per minute increasing to 3 L per minute if necessary) can be controlled on demand over the standard air valve. Air supply with the Pentax processor or CO_2_ insufflation with the CO_2_ Efficent Insufflator cannot be acoustically discriminated. The valves for the endoscope using CO_2_ are identical with the standard valves. Moreover, the switch for the pump of the CO_2_ processor was hidden as was the switch for the insufflator. The front line of the processor and the CO_2_ insufflator were covered to mask the operational status. The setup was done by the assisting nurse prior to the start of the colonoscopy without knowledge of the examiner. Therefore, this was a truly double-blinded trial. All patients were sedated with propofol using standard procedures previously described [14]. A level of conscious sedation (now classified as moderate sedation) was targeted giving propofol boli of 10 or 20 mg according to ASA-Classification. The total dose of propofol was registered and used for later analysis.

The primary endpoint of the present investigation was overall pain scores. Secondary endpoints were bloating as well as overall satisfaction assessed on a 10-point visual analogue scale (VAS) before and after the examination at 1, 3, 6, and 24 hours. 

The grading of the VAS were as follows: for pain: 0 = no pain at all, 10 = worst possible pain; for bloating: 0 = no bloating at all, 10 = terrible bloating; for satisfaction: 0 = not at all satisfied, 10 = perfectly satisfied.

As quality indicators and characteristics of the performed colonoscopy time to reach the ileum, withdrawal time, length of intervention, procedural length, and the number and the size (>1 cm, <1 cm, small polyp <5 mm) of removed polyps and their histology were assessed. Finally, periprocedural complications were registered as well as time to discharge, propofol dosage, oxygen supply, and other medication given. We also continuously measured transcutaneous carbon dioxide with a Sentec Capnograph device (SenTec AG 4106 Therwil, Switzerland) for respiratory monitoring.

The patients were motivated at discharge to fill out the postprocedure survey regarding pain, bloating, and satisfaction. We purposefully and a priori decided not to call any patients who did not mail in the survey, as this would have potentially led to imprecise results from patients retrospectively filling out the survey. 

### 2.1. Statistics

Statistical analyses were performed using SPSS Version 11.0, and the level of statistical significance was set at *P* < .05. To compare dichotomous (yes/no) and categorical outcomes, the Chi Square test was used. For comparisons of continuous outcomes, unpaired *t*-tests were used.

The sample size computations were based on a difference for overall pain scores (the primary endpoint) with an effect size of 2 (overall pain score of 4 in the CO_2_ group versus 6 in the room air group). With a power of 80% (beta error of 20%), type I error of 5%, and standard deviation of 5, the resulting sample size was 200 patients.

## 3. Results

A total number of 219 patients undergoing colonoscopy were enrolled in this study. One hundred and ten patients were randomized to the CO_2_ group and 109 to the room air group. The baseline characteristics of both groups are displayed in Table 1. These baseline characteristics were similar in both groups with the exception of gender and the fraction of patients with irritable bowel syndrome: there were significantly more women (62% versus 47%, *P* = .03) and IBS patients in the CO_2_ group (Table 1).

The duration to reach the ileum was 7.7 ± 4.7 minutes in the CO_2_ group and 6.7 ± 4.1 in the room air group (*P* > .05). Withdrawal times were 13.6 ± 6.0 minutes in the CO_2_ group and 13.3 ± 6.1 minutes in the room air group (*P* > .05). Outcomes are listed in Table 2. In 10.9% of the patients in the CO_2_ group, relevant polyps were removed (relevant polyps were defined as by size over 1 cm, histology (e.g., villous and serrated) or by high grade dysplasia). In the air group, the corresponding percentage was 7.3%. Additional adenomas (smaller than 1 cm) were found in 34.5% versus 33.9%, respectively (*P* > .05).

For the data collection in the office, data was complete for all patients. Seventy-five percent of group 1 and 82% of group 2 returned the completed questionnaire. 

**Figure 1:**
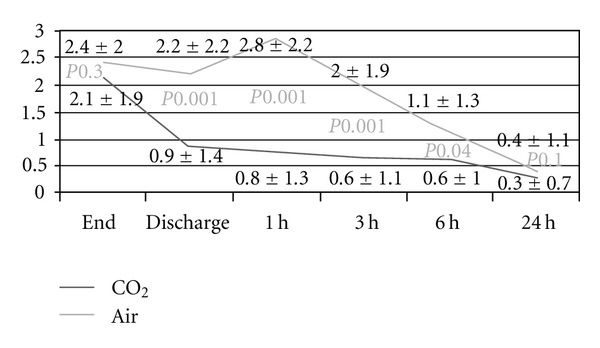
Bloating over time depicted as visual analogue scale scores.

**Figure 2:**
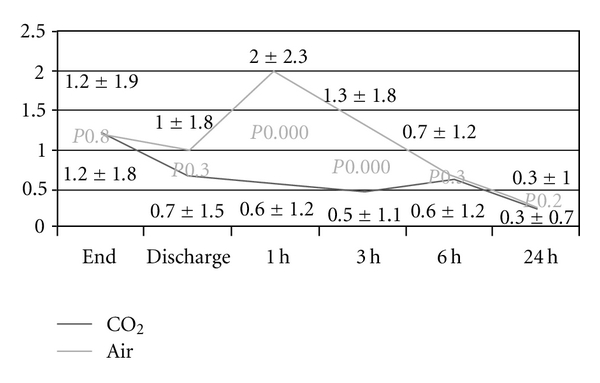
Pain over time depicted as visual analogue scale scores.

At all time points, the VAS scores for bloating and pain were lower in the CO_2_ group compared to the room air group (Figures 1 and 2): patients experienced significantly less bloating at discharge, as well as one hour (*P* < .001), three hours (*P* < .001), and six hours after the procedure (*P* = .04). Also, patients randomized to CO_2_ experienced significantly less pain at one hour (*P* < .001) and three hours (*P* < .001) after the procedure. Overall pain summary scores for 24 hours were significantly lower in the CO_2_ group compared to the patients assigned to room air (3.6 ± 5.8 versus 6.1 ± 7.4, *P* = .014). Similarly, overall pain scores were significantly lower in patients randomized to CO_2_ versus room air (2.0 ± 3.8 versus 4.0 ± 5.0, *P* = .007). The percentage of patients, who did not experience any pain, was significantly higher in the group examined with CO_2_ up to 3 hours (Table 3). Bloating was also significantly less in the above-mentioned time periods (*P* values between .023 to <.001). 

The overall acceptance of the colonoscopy was excellent in both groups. In the CO_2_ group, the overall satisfaction score was 9.6 ± 0.7 versus 9.3 ± 1.0 on a 10-point visual analogue scale.

**Figure 3:**
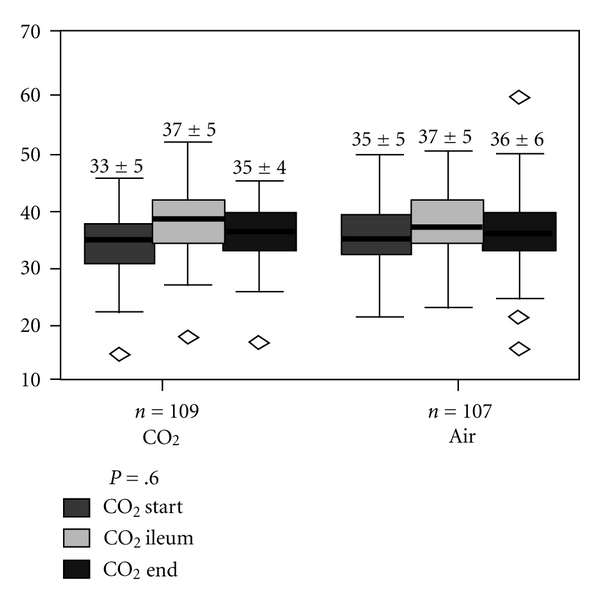
Transcutaneously measured CO_2_ values in CO_2_ and room air group.

CO_2_ was continuously measured. There was no significant difference with respect to transcutaneous CO_2_ levels between the CO_2_ and room air groups (pCO_2_ at the end of procedure in CO_2_ group: 35.7 ± 4.3 mmHg versus room air group: 35.8 ± 6.5 mmHg in group 2, Figure 3). In both groups, there was a slight increase in CO_2_ from baseline to the ileum; however, the CO_2_ values remained within normal range. In all patients, including the known COPD patients, the O_2_ saturation always stayed above 88% and no ventilation, manual, or mechanical airway assistance was required.

## 4. Discussion

This represents the first large double-blinded randomized controlled trial comparing the use of CO_2_ versus room air in patients undergoing colonoscopy with propofol sedation using continuous monitoring by capnography for all patients. Our investigation provides compelling evidence that CO_2_ insufflation compared with standard room air significantly reduces bloating and pain in patients undergoing routine colonoscopy and stands in line with the review reported by Dellon et al. [11] and several other studies [9, 10, 15]. More important, the procedure is safe with no significant differences in CO_2_ measurements observed between the two groups. This is of utmost importance, since data are limited on this topic with safety still being debated in sedated patients.

The setting of the present investigation has several strengths. First, this study has a large sample size, notably not selected without any exclusion, neither for pulmonary disorders or former abdominal surgery. This makes it possible to transfer our data to a screening population without any restrictions (not even for COPD). Second, it is one of the few [10] studies comparing CO_2_ and room air use in a double-blinded randomized fashion. Third, all patients were sedated, and the CO_2_ group was continuously monitored. Fourth, only one endoscopist performed all procedures, which removes technical skills as a potential confounder. Fifth, the use of CO_2_ for routine colonoscopy was associated not only with significantly less pain and bloating but also with superior patient tolerance. This benefit is particularly remarkable considering that the CO_2_ group had significantly more women and more IBS patients compared to the room air group. Of note, CO_2_ benefits are observed immediately after the sedation. The pain reducing effect seems is most apparent and profound in the first 3 hours after the procedure, an observation that was also seen in other studies [10]. Thereafter, a tendency of lower pain perception persists. Stevenson et al. [16] showed a persisting benefit even at 24 hours. The duration of this benefit might vary depending on the amount of air inflated (which is endoscopist dependent), the examination time, and the interventions, making it most valuable for patients with large polyp resections. 

Finally, the use of CO_2_ was not associated with a prolonged preparation time to set up or perform the procedure, and there were no side effects and no complications. 

Abdominal pain after colonoscopy is common and distressing for some patients and can cause even sick leave [13]. It is due to bowel distension by the insufflating gas [12]. CO_2_ insufflation, as commonly used for establishing pneumoperitoneum during laparoscopic surgery, has already been proposed and introduced in different fields of endoscopy [17–19]. This procedure has a potential of resulting in less periprocedural pain, especially since new and easy to use insufflators are on the market. Increasingly sophisticated endoscopic procedures are currently being developed and performed. Some of them are time consuming such as endoscopic submucosal dissection (ESD) or therapeutic endoscopic retrograde cholangiopancreaticography (ERCP). Interestingly, an advantage for CO_2_ use was demonstrated in patients undergoing ERCP as well as ESD with respect to periprocedural pain [17, 18]. In a recently published trial by Domagk et al. [19], it was shown that CO_2_ use for balloon enteroscopy was not only less painful for patients, but also associated with a significantly deeper intubation length of the small intestine. Another advantage is that CO_2_ is less combustible in presence of stool or sub-optimal bowel preparation and, therefore, potentially safer when diathermy is being used. Moreover, it has advantages in longer procedures to avoid overdistention of the colon. However, the number of studies investigating the use of CO_2_ is limited and safety concerns with respect to respiratory side effects have been raised. This issue was assessed in three studies. The first including nonsedated patients undergoing colonoscopy showed a slight, however, clinically irrelevant (CO_2_ values within normal range) increase in end-tidal pCO_2_ with CO_2_ insufflation compared to room air use [20]. No difference in pCO_2_ was reported in the study of Yamano et al. [10] in nonsedated patients. In another small investigation, no difference was found between partially sedated patients undergoing colonoscopy with room air versus CO_2_. The authors concluded that CO_2_ insufflation was safe [21]. However, this investigation included only 29 sedated patients undergoing colonoscopy with the use of CO_2_. Here, we show the safety of CO_2_ insufflation in a large sample of sedated patients. 

It is well known and intuitive that the success of any screening strategy is critically dependent on population acceptance of the screening methods; efforts to minimize discomfort associated with colonoscopy may positively influence compliance. With the prospect of widespread colonoscopy screening for colorectal cancer in asymptomatic populations, it is imperative to optimize patient comfort and convenience as well as the quality of the examination itself. Based on the findings of the present double-blinded, randomized controlled trial, CO_2_ appears to be important to reach this goal with less bloating, less pain, and higher patient satisfaction. The lack of awareness [1] of this compelling technique should be changed over time, as endoscopists are more and more convinced by growing evidence of the usefulness and safety of CO_2_ insufflation.

## 5. Strengths and Limitations

We would like to acknowledge the limitations of this study. First, despite the randomized controlled study design, there were some imbalances between the CO_2_ and room air group with respect to gender and IBS. In fact, the CO_2_ group contained more female and IBS patients. However, it is well known that IBS and female patients usually have more pain and bloating after colonoscopy [22, 23]. Therefore, this will bias our findings towards the null hypothesis and the true benefit of CO_2_ colonoscopy may be even more important. Second, the randomization was done with a dice for each patient, and thus is theoretically prone to bias. However, a study nurse did the randomization without any involvement of the investigators, and therefore, the risk of bias is minimal. 

There are several strengths of this study: first, this investigation was done in a double-blinded randomized, controlled fashion. Second, this is a large randomized patient sample; more important, there are only few reports that have investigated the benefit of CO_2_ in sedated patients with continuous CO_2_ monitoring. Third, there were no exclusion criteria in this study, and therefore, the generalizability (external validity) is high. Most importantly, the present trial addresses a very relevant and novel research questions and hopefully will help changing practice patterns.

## 6. Conclusion

This study provides compelling evidence that CO_2_ insufflation is associated with significantly less bloating and pain during and after routine colonoscopy. Colonoscopy with CO_2_ insufflation is safe as no significant differences in CO_2_ measurements were observed. Based on these data, the routine use of CO_2_ insufflation for colonoscopy is encouraged.

## Figures and Tables

**Table 1:** Baseline patient characteristics.

	CO_2_ group *n* = 110	Room air group *n* = 109	*P* value
Age	58 ± 13	62 ± 12	.42
Sex	Female: 62%	Female: 47%	.03
BMI	25 ± 4.7	26 ± 4.4	.78

Comorbidity			.36
ASA I	64.5%	61.5%	
ASA II	29.1%	26.6%	
ASA III	6.4%	11.9%	
COPD	2.7%	2.8%	.32

Irritable bowel syndrome	23.6%	12.8%	.03

Indication			.92
Screening	78.2%	79.9%	
Surveillance	3.6%	4.6%	
IBD	6.4%	4.6%	

**Table 2:** Parameters of colonoscopy in CO_2_ and room air group.

	CO_2_ group	Room air group	*P* value
Propofol	134 mg ± 56	120 mg ± 120	.9
Nasal oxygen substitution if O_2_ saturation <90%	10%	20.2%	.02
Ileum intubation rate	95.5%^1^	97.2% ^1^	.22
Time to ileum	7.7 ± 4.7 min	6.7 ± 4.1 min	.18
Withdrawal time	13.6 ± 6.0 min	13.3 ± 6.1 min	.99
Intervention time	3.3 ± 4.5 min	3.2 ± 4.6 min	.99

Polyps	68.2%	68.8%	.11
>1 cm	8.2%	7.3%	
0.5–1 cm	17.3%	16.6%	
<0.5 cm	63.6%	66.1%	

Findings			.77
Carcinoma	0.9%	1.8%	
Relevant polyp^2^	10.9%	7.3%	
Small adenoma^3^	34.5%	33.9%	
Hyperplastic	13.6%	14.7%	

CO_2_ (mmHg)			
at start	33.4 ± 4.7	34.6 ± 5.1	.59
at ileum	37.3 ± 5.2	37.0 ± 5.2	.62
end of examination	35.2 ± 4.3	35.6 ± 6.0	.01
Maximal CO_2_ rise	4.2 ± 3.7	2.9 ± 4.4	.77

^1^0.9% stenosis (e.g., tumor, or sigmoid stenosis due to diverticulosis) in both groups, reaching coecum in 99% in both groups.

^2^Relevant polyps defined as polyp >1 cm, serrated, and villous ± high-grade dysplasia.

^3^Polyps size <1 cm.

**Table 3:** Pain sensation assessed by VAS Score.

Time	Insufflation	% (*n* = absolute numbers)				*P* value
		VAS 0	VAS 1-2	VAS 3–5	VAS 6–10	
End	CO_2_	59 (65)	24.5 (27)	14.5 (16)	2 (2)	.94
	Air	57.7 (63)	23 (25)	16.5 (18)	2.8 (3)	

Discharge	CO_2_	73.6 (81)	14.5 (16)	10.9 (12)	0.1 (1)	.23
(15–30 min)	Air	64.2 (70)	22 (24)	8.3 (9)	5.5 (6)	

1 h	CO_2_	68.8 (55)	22.5 (18)	8.8 (7)	0	<.0001
	Air	36.4 (32)	34 (30)	18.2 (16)	11.4 (10)	

3 h	CO_2_	72.2 (57)	21.5 (17)	6.3 (5)	0	.015
	Air	51.1 (45)	28.4 (25)	14.8 (13)	5.7 (5)	

6 h	CO_2_	73.8 (59)	20 (16)	6.2 (5)	0	.53
	Air	66 (58)	22.7 (20)	10.2 (9)	1.1 (1)	

24 h	CO_2_	87.5 (70)	7.5 (6)	5 (4)	0	.67
	Air	86.4 (76)	11.4 (10)	2.3 (2)	0	
